# Strategies for Cardiovascular Disease Prevention in Type 1 Diabetes: A Comprehensive Review

**DOI:** 10.7759/cureus.66420

**Published:** 2024-08-08

**Authors:** Abhinav Ahuja, Sachin Agrawal, Sourya Acharya, Venkat Reddy, Nitish Batra

**Affiliations:** 1 Internal Medicine, Jawaharlal Nehru Medical College, Datta Meghe Institute of Higher Education and Research, Wardha, IND

**Keywords:** integrated care models, pharmacological interventions, lifestyle modifications, risk assessment, type 1 diabetes, cardiovascular disease prevention

## Abstract

Cardiovascular disease (CVD) is a leading cause of morbidity and mortality among individuals with type 1 diabetes (T1D), necessitating effective prevention strategies. This comprehensive review consolidates current knowledge and evidence on preventing CVD in T1D patients. It begins by exploring the pathophysiological mechanisms that link T1D to an increased risk of CVD, highlighting factors such as chronic hyperglycemia, hypertension, dyslipidemia, and inflammation. The review also examines the epidemiology and specific risk factors for CVD in this population, emphasizing the need for rigorous risk assessment and screening. Lifestyle modifications, including dietary interventions, regular physical activity, and smoking cessation, are evaluated for their effectiveness in reducing CVD risk. Additionally, the review discusses pharmacological interventions, such as insulin therapy for glycemic control, antihypertensive medications, lipid-lowering agents, and antiplatelet therapy, underscoring their critical role in CVD prevention. Emerging therapies and future research directions are explored, focusing on novel pharmacological agents, advances in insulin delivery systems, and personalized medicine approaches. The importance of integrated care models involving multidisciplinary teams and the use of technology is highlighted as essential for comprehensive management. Challenges and barriers to implementing these strategies, including healthcare system limitations, patient adherence, and socioeconomic factors, are also addressed. This review provides a detailed synthesis of current strategies and future directions for preventing CVD in individuals with T1D, serving as a valuable resource for clinicians, researchers, and policymakers dedicated to improving cardiovascular outcomes in this high-risk population.

## Introduction and background

Type 1 diabetes (T1D) is a chronic autoimmune condition characterized by the destruction of insulin-producing beta cells in the pancreas, leading to an absolute deficiency of insulin [1]. This condition requires lifelong administration of exogenous insulin to maintain glycemic control. Unlike type 2 diabetes, which is often associated with insulin resistance and is more prevalent in adults, T1D typically manifests in childhood or adolescence, although it can develop at any age [2]. The exact causes of T1D remain unclear, involving a complex interplay of genetic predisposition and environmental triggers. Managing T1D is intricate, necessitating regular blood glucose monitoring, dietary regulation, and physical activity to prevent acute and chronic complications [1].

Cardiovascular disease (CVD) represents one of the most critical long-term complications for individuals with T1D. Patients with T1D face a heightened risk of developing various forms of CVD, including coronary artery disease, cerebrovascular disease, and peripheral artery disease. This increased risk stems from multiple factors, such as chronic hyperglycemia, hypertension, dyslipidemia, and a pro-inflammatory state, all of which accelerate the development of atherosclerosis [3]. The impact of CVD in T1D patients is profound, significantly affecting their quality of life and contributing to increased morbidity and mortality. Consequently, effective strategies for the prevention and management of CVD are essential to mitigate its burden on the T1D population [4].

The primary objective of this comprehensive review is to collate and synthesize the current knowledge and evidence on strategies for preventing CVD in individuals with T1D. This review aims to elucidate the pathophysiological mechanisms that link T1D to an elevated risk of CVD and discuss the epidemiology and risk factors associated with CVD in T1D patients. Furthermore, it seeks to evaluate current guidelines and recommendations for CVD risk assessment and screening in T1D, analyze the effectiveness of various lifestyle modifications and pharmacological interventions in reducing CVD risk, and explore emerging therapies and future directions in preventing CVD in T1D. Additionally, the review highlights the importance of integrated care models and the role of multidisciplinary teams (MDTs) in managing T1D and preventing CVD while identifying challenges and barriers to implementing these strategies in clinical practice.

## Review

Pathophysiology of CVD in T1D

Chronic hyperglycemia is a central factor in the pathogenesis of CVD in individuals with T1D. Prolonged elevated blood glucose levels contribute to the development of microvascular complications, such as nephropathy and neuropathy, which are significant risk factors for CVD. Research indicates that even a 1% increase in glycated hemoglobin (HbA1c) correlates with a substantial increase in coronary vessel stenosis, underscoring the direct impact of glycemic control on cardiovascular health [5]. Moreover, elevated glucose levels lead to non-enzymatic glycosylation of proteins, forming advanced glycation end-products (AGEs). These AGEs promote vascular damage and atherosclerosis by enhancing inflammation and oxidative stress. Additionally, fluctuations in blood glucose levels, known as glucose variability, can further elevate cardiovascular risk by inducing oxidative stress and vascular inflammation, even in patients with well-controlled HbA1c levels [6].

Inflammation plays a crucial role in the development of CVD in T1D. The condition is characterized by chronic inflammation, which contributes to endothelial dysfunction. Inflammatory markers, such as C-reactive protein (CRP), are often elevated in individuals with T1D, indicating an ongoing inflammatory response that can lead to vascular injury and atherosclerosis [7]. Endothelial dysfunction, marked by reduced availability of nitric oxide (NO), increased vascular permeability, and enhanced monocyte adhesion to the endothelium, is a precursor to atherosclerosis and can significantly impair vascular health. This dysfunction facilitates the development of plaques in the arteries and increases the risk of acute cardiovascular events [7].

**Figure 1:**
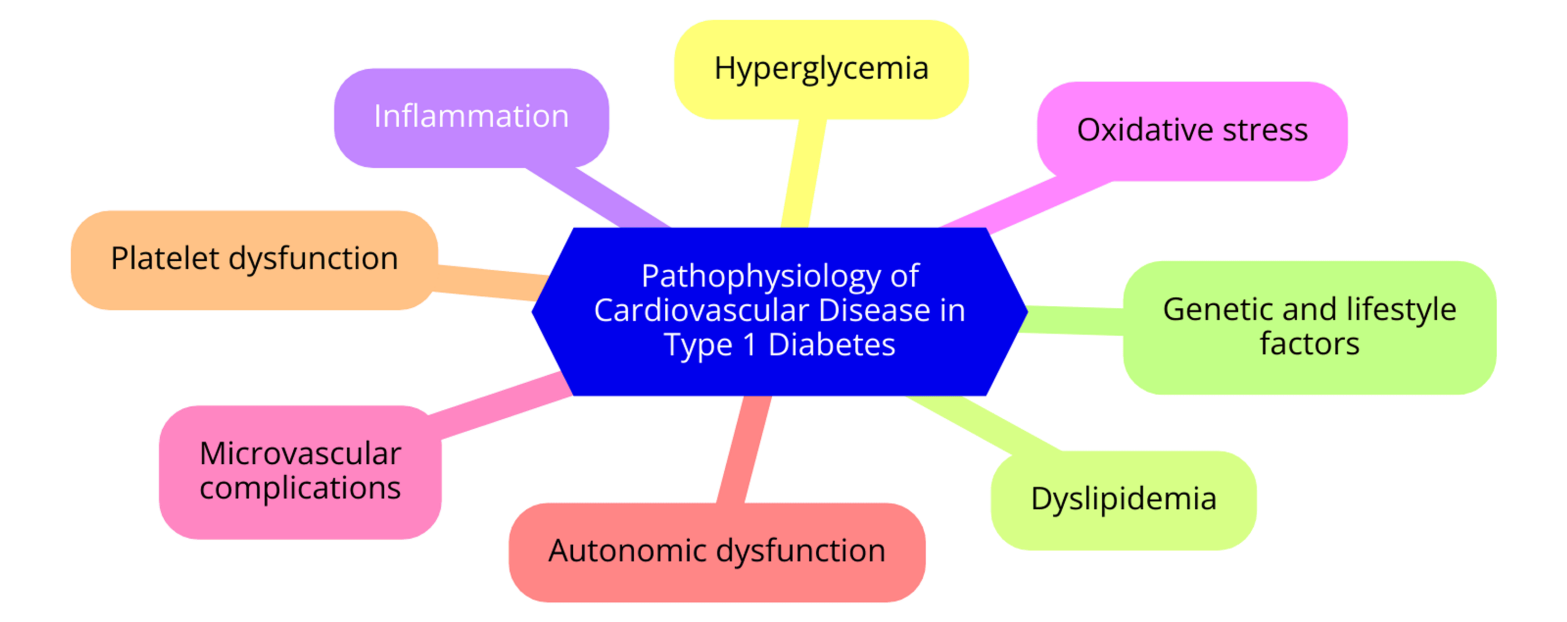
The pathophysiology of cardiovascular disease in type 1 diabetes Image credit: Dr. Abhinav Ahuja

Oxidative stress is another significant contributor to the cardiovascular complications associated with T1D. Chronic hyperglycemia stimulates the production of reactive oxygen species (ROS) through the mitochondrial electron transport chain, leading to oxidative damage in vascular tissues. This oxidative stress can harm endothelial cells, promote inflammation, and accelerate atherosclerosis [8]. Furthermore, mitochondrial dysfunction resulting from hyperglycemia-induced oxidative stress can create a vicious cycle of damage that exacerbates cardiovascular risk. Even in well-controlled T1D patients, qualitative and functional abnormalities of lipoproteins have been observed, which may be influenced by oxidative stress and contribute to the development of atherosclerosis [8]. The pathophysiology of CVD in T1D is shown in Figure 1.

Epidemiology of CVD in T1D

Prevalence of CVD in T1D Patients

The prevalence of CVD is significantly higher in individuals with T1D compared to the general population. Research indicates that the prevalence of CVD among T1D patients ranges from approximately 6% in younger individuals aged 15-29 years to around 25% in those aged 45-59 years. This stark increase underscores the impact of age and the duration of diabetes on cardiovascular health [9]. CVD tends to manifest earlier in life for individuals with T1D, with a notable correlation between the duration of diabetes and the risk of developing cardiovascular complications. Studies have shown that the overall mortality risk associated with CVD in T1D patients is two to three times higher in men and three to five times higher in women compared to their non-diabetic counterparts. This elevated risk highlights the critical need for targeted prevention strategies [4]. Moreover, CVD is the leading cause of death among individuals diagnosed with T1D before the age of 18, particularly after 20 years of living with the condition. This emphasizes the long-term cardiovascular risks accompanying T1D, necessitating vigilant monitoring and management throughout the patient's life [10]. In terms of incidence, studies have reported that the rate of major coronary artery disease events in young adults (aged 28-38 years) with T1D is approximately 1% per year. This statistic further illustrates the heightened cardiovascular risk faced by younger individuals with T1D, reinforcing the importance of early intervention and lifestyle modifications to mitigate these risks [11].

Risk Factors for CVD in T1D

**Figure 2:**
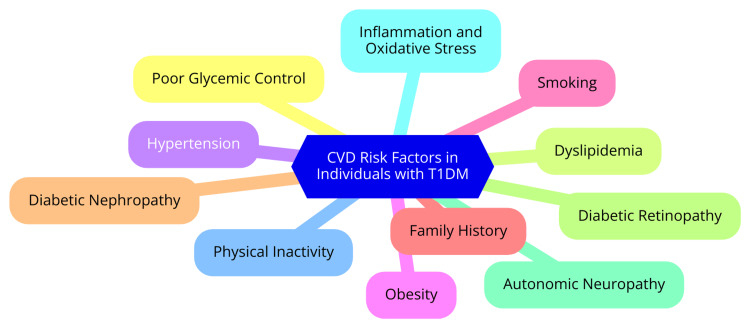
Cardiovascular disease (CVD) risk factors in individuals with type 1 diabetes mellitus (T1DM) Image credit: Dr. Abhinav Ahuja

CVD poses a significant threat to individuals with T1D, driven by a range of interconnected risk factors. Understanding these risk factors is crucial for implementing effective prevention and management strategies to mitigate this population's heightened risk of CVD [12]. One of the primary risk factors for CVD in T1D is hyperglycemia. Chronic high blood glucose levels are strongly linked to cardiovascular complications. Research indicates that for every percentage point increase in HbA1c, the risk of major atherosclerotic cardiovascular events (MACE) increases by 31% for any CVD and 42% for MACE specifically. This underscores the importance of maintaining optimal glycemic control to reduce cardiovascular risk [13]. Age is another significant factor influencing CVD risk in T1D patients. The likelihood of experiencing cardiovascular events increases with age, particularly for those diagnosed with diabetes at a younger age. Additionally, blood pressure plays a critical role; hypertension is prevalent among T1D patients and is associated with a higher risk of cardiovascular complications. Studies have shown that higher mean systolic blood pressure correlates with increased risks of both CVD and MACE [14]. Lipid abnormalities, particularly dyslipidemia, characterized by elevated low-density lipoprotein cholesterol (LDL-C) and triglycerides, further contribute to cardiovascular risk in individuals with T1D. These lipid profile abnormalities can significantly heighten the risk of cardiovascular events over time [15]. Moreover, the duration of diabetes is a crucial factor; the longer an individual has had T1D, the greater the risk of developing cardiovascular complications. Traditional risk factors, such as hypertension and dyslipidemia, often emerge several years after the diagnosis of T1D, with their impact becoming more pronounced after 15 to 20 years [15]. Lifestyle factors also play a vital role in increasing cardiovascular risk. Smoking, physical inactivity, and poor dietary habits can exacerbate the risk of CVD, making these modifiable factors critical targets for intervention [16]. Furthermore, a family history of CVD can elevate an individual's risk, suggesting a potential genetic predisposition [16]. Emerging research highlights the influence of recurrent hypoglycemia and glucose variability on cardiovascular risk in T1D patients. These factors may independently contribute to cardiovascular complications, complicating this population's traditional understanding of risk [17]. CVD risk factors in individuals with type 1 diabetes mellitus (T1DM) are shown in Figure 2.

Gender and Age Disparities

CVD presents notable gender and age disparities among individuals with T1D. Research indicates that women with T1D face a higher relative risk for CVD compared to their male counterparts, which contrasts with the general population, where men typically exhibit a greater risk [18]. This heightened vulnerability in women may be attributed to several factors, including differences in body fat distribution. Women with T1D often have more centrally distributed fat, which is associated with increased cardiovascular risk. Furthermore, the protective cardiovascular effects usually enjoyed by premenopausal women diminish in the presence of T1D, leading to an elevated risk of CVD at a younger age [19]. The mortality rates related to CVD are particularly concerning for women with T1D. Studies have shown that these women experience higher cardiovascular mortality rates even in the absence of a previous CVD history compared to women without diabetes who have had cardiovascular events [20]. Additionally, women with T1D tend to exhibit more cardiovascular risk factors, such as higher pulse rates and HbA1c levels, compared to men, despite men generally having less favorable profiles for some risk factors. This suggests that gender-specific factors may influence the development and progression of CVD in women with T1D [20]. Age also plays a critical role in the risk of CVD among T1D patients. CVD tends to manifest at a younger age and with greater prevalence in individuals with T1D compared to the general population. As patients age and the duration of diabetes increases, the risk of developing CVD escalates [21]. The Diabetes Control and Complications Trial (DCCT) and its follow-up Epidemiology of Diabetes Interventions and Complications (EDIC) study highlighted that while there are no significant differences in age, body mass index (BMI), or HbA1c levels between sexes among those who develop CVD, the overall risk still varies significantly by gender, particularly at younger ages [21].

Risk Assessment and Screening

CVD remains the leading cause of morbidity and mortality in individuals with T1D. While significant progress has been made in reducing CVD mortality in T1D patients over the past few decades, the overall risk of CVD is still two to five times higher compared to non-diabetic individuals, particularly in women. Preventing cardiovascular complications in T1D requires a multifactorial approach targeting various risk factors [22]. Individuals with T1D have a two to five times higher risk of CVD compared to the general population. However, recent studies suggest that not all T1D patients are at equally high risk. Age, diabetes duration, and other risk factors or complications can stratify T1D patients into different risk categories [23]. Specific risk assessment tools have been developed for individuals with T1D to evaluate CVD risk more accurately than general population tools. Notable tools include the Swedish National Diabetes Register (NDR) and the Steno Risk Engine. The frequency of cardiovascular risk assessment in T1D patients should be tailored based on individual risk factors, with annual screening for most adults and increased frequency for high-risk patients [23]. Chronic hyperglycemia is central in developing microvascular and macrovascular complications in T1D. Intensive insulin therapy has been shown to reduce the occurrence and progression of both microvascular and macrovascular complications, as evidenced by the DCCT/EDIC study. Maintaining optimal glycemic control, measured by HbA1c, is crucial for preventing CVD in T1D patients [24]. In addition to hyperglycemia, other modifiable risk factors for CVD in T1D include hyperlipidemia, overweight/obesity, hypertension, and smoking. Screening for and managing these risk factors is essential for reducing CVD risk. Lifestyle modifications, such as a healthy diet, regular exercise, and smoking cessation, should be encouraged. When lifestyle changes alone are insufficient, pharmacological interventions may be necessary to manage cardiovascular risk factors [25]. Regular screening for subclinical CVD, such as coronary artery calcification and cardiac autonomic neuropathy, is important for early detection and intervention. Screening should begin in adolescence and continue throughout adulthood, with frequency based on the patient's age and risk profile [26]. Effective prevention of CVD in T1D requires a multidisciplinary approach involving endocrinologists, cardiologists, dietitians, and other healthcare professionals. Regular follow-up and care coordination are essential for addressing all aspects of cardiovascular risk management [26].

Lifestyle modifications

Dietary interventions are fundamental in managing cardiovascular health for individuals with T1D. A heart-healthy diet is characterized by low saturated fat, trans fat, and cholesterol, and it is rich in soluble fiber and antioxidants. This diet emphasizes consuming legumes, oats, berries, nuts, leafy greens, and fatty fish, all contributing to lowering cardiovascular risk. Among various dietary patterns, the Mediterranean diet stands out for its beneficial effects [27]. This diet focuses on whole foods, including fruits, vegetables, whole grains, fish, and healthy fats, particularly olive oil. Research has shown that adherence to the Mediterranean diet can significantly reduce cardiovascular risk factors in people with diabetes, making it an excellent choice for those with T1D [27]. Another dietary approach that has gained attention is the low-carbohydrate diet. While this diet can effectively manage blood glucose levels, it is crucial to ensure that individuals still consume adequate amounts of fruits, vegetables, and whole grains to meet their nutritional needs [28]. A balanced intake of these foods provides essential nutrients and fiber, vital for maintaining heart health. Therefore, individuals with T1D should work closely with healthcare providers or dietitians to develop a personalized eating plan that supports glycemic control and cardiovascular health [28].

Physical activity is another cornerstone of CVD prevention in individuals with T1D. At least 150 minutes of moderate-intensity aerobic exercise each week is recommended to improve cardiovascular fitness and insulin sensitivity. Activities, such as brisk walking, cycling, swimming, or dancing, can be enjoyable and effective ways to meet this goal. Regular exercise not only aids in weight management but also contributes to better blood pressure control and improved lipid profiles, all of which are essential for reducing cardiovascular risk [29]. The benefits of regular exercise extend beyond physical health; they also encompass mental well-being. Exercise has been shown to reduce stress, anxiety, and depression, which can further improve overall health outcomes. For individuals with T1D, incorporating physical activity into their daily routine can lead to a more balanced lifestyle and help mitigate the risks associated with CVD [30].

Smoking cessation is a critical lifestyle modification for reducing CVD risk, particularly for individuals with T1D. Tobacco use significantly increases the likelihood of cardiovascular events, compounding the already elevated risk faced by those with diabetes. Quitting smoking can lead to substantial improvements in cardiovascular health, making it one of the most impactful changes a person can make [31]. To support individuals in their efforts to quit smoking, various strategies can be employed. These may include behavioral therapy, nicotine replacement therapies, and support from healthcare providers or smoking cessation programs. Encouragement and resources tailored to individual needs can significantly enhance the chances of successfully quitting. By prioritizing smoking cessation, individuals with T1D can take a significant step toward improving their cardiovascular health and overall quality of life [32].

Pharmacological interventions

Glycemic control is a cornerstone of diabetes management, particularly for individuals with T1D, as it significantly impacts both short-term and long-term health outcomes. Insulin therapy is essential for these patients due to the absence of endogenous insulin production [33]. Most individuals benefit from intensive insulin management, which typically involves multiple daily injections of prandial (rapid-acting) and basal (long-acting) insulin or the use of continuous subcutaneous insulin infusion (CSII). This approach has been shown to improve glycemic control significantly, reducing HbA1c levels and lowering the risk of both microvascular and macrovascular complications. In addition to insulin therapy, continuous glucose monitoring (CGM) systems have emerged as a valuable tool for managing T1D [34]. CGM devices provide real-time glucose readings, enabling patients to track their glucose levels continuously throughout the day. This technology allows for better glycemic control and reduces the risk of hypoglycemia. By providing insights into glucose trends and patterns, CGM facilitates timely insulin therapy and dietary intake adjustments, empowering patients to manage their diabetes more effectively [35]. Effective blood pressure management is critical in individuals with T1D, as hypertension significantly increases the risk of CVD. Antihypertensive medications, particularly angiotensin-converting enzyme (ACE) inhibitors and angiotensin II receptor blockers (ARBs), are commonly prescribed to control blood pressure [36]. These medications help achieve target blood pressure levels and provide renal protection, especially in patients with diabetic nephropathy. The recommended target blood pressure for adults with T1D is less than 140/90 mmHg. Achieving and maintaining this target is essential for minimizing cardiovascular risks and protecting renal function. Regular monitoring of blood pressure and adjustments to antihypertensive therapy is necessary to ensure that patients remain within these optimal ranges [37].

**Figure 3:**
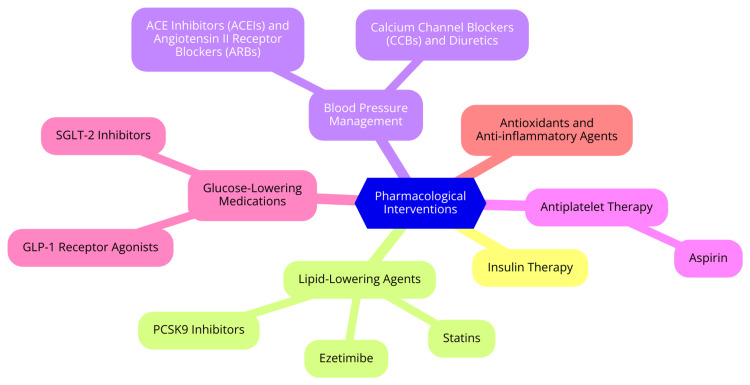
Pharmacological interventions Image credit: Dr. Abhinav Ahuja

Lipid management is another crucial component of CVD prevention in T1D. Statins are the cornerstone of this management strategy, particularly for patients over 40 years old or those with additional cardiovascular risk factors. Statins have been shown to effectively reduce low-density lipoprotein (LDL) cholesterol levels and lower the incidence of cardiovascular events in this population [38]. In some cases, other lipid-lowering agents, such as ezetimibe and proprotein convertase subtilisin/kexin type 9 (PCSK9) inhibitors, may be considered for patients who do not achieve target lipid levels with statins alone. For individuals with T1D, the target lipid levels include an LDL cholesterol level of less than 100 mg/dL and a total cholesterol level of less than 200 mg/dL. Regular lipid profiling is recommended to monitor levels and adjust treatment as necessary, ensuring patients maintain optimal lipid profiles to reduce cardiovascular risk [39]. Antiplatelet therapy, particularly with aspirin, may be indicated for individuals with T1D who have a high risk of cardiovascular events. This includes patients with established CVD or those exhibiting multiple risk factors. The decision to initiate aspirin therapy should be individualized based on the patient’s overall risk profile, considering the potential benefits and risks associated with its use [40]. The benefits of antiplatelet therapy include a reduction in the risk of myocardial infarction and stroke, which are significant concerns for individuals with diabetes. However, there are associated risks, such as gastrointestinal bleeding and hemorrhagic stroke. Therefore, carefully considering and discussing the risks versus benefits are essential before starting antiplatelet therapy, ensuring that patients are well-informed and involved in their treatment decisions [41]. Pharmacological interventions are shown in Figure 3.

Emerging therapies and future directions

Recent advancements in pharmacological research have introduced several novel agents to preserve beta-cell function and enhance insulin secretion in individuals with T1D. One promising candidate is verapamil, a medication traditionally used for hypertension, which has shown potential in maintaining beta-cell function by reducing intracellular calcium levels [42]. This action helps inhibit processes that lead to beta-cell apoptosis, and clinical trials have indicated that verapamil can sustain C-peptide levels while reducing insulin requirements in newly diagnosed T1D patients [42]. Another agent under investigation is gamma-aminobutyric acid (GABA), a neurotransmitter that may protect beta cells by promoting membrane hyperpolarization and enhancing cell survival and function. Additionally, tauroursodeoxycholic acid (TUDCA), known for its cytoprotective properties, is being explored for its ability to protect pancreatic beta cells from dysfunction and apoptosis. Furthermore, glucagon-like peptide-1 (GLP-1) receptor agonists, typically used in type 2 diabetes, are being studied for their potential to enhance insulin secretion and provide protective effects on beta cells in T1D patients [42]. Innovations in insulin delivery systems are crucial for improving glycemic control and enhancing patient adherence in the management of T1D. CGM systems have revolutionized diabetes care by providing real-time glucose readings and enabling more precise insulin dosing. When integrated with insulin pumps, CGMs facilitate automated insulin delivery, significantly improving glycemic control and reducing the risk of hypoglycemic events. Another advancement is the development of smart insulin pens, which track insulin doses and provide reminders to patients, helping them manage their diabetes more effectively. Moreover, closed-loop systems, often called artificial pancreas systems, combine insulin pumps with CGMs to automate insulin delivery based on real-time glucose levels. This technology enhances patient convenience and control, allowing for more seamless diabetes management [43].

Ongoing genetic and molecular research is providing valuable insights into the pathophysiology of T1D and identifying potential therapeutic targets. For instance, studies on the PAX4 gene, involved in beta-cell development, suggest that targeting its pathways could promote beta-cell proliferation and survival in T1D [44]. Additionally, researchers are investigating DYRK1A inhibitors, which may enhance beta-cell proliferation, offering a potential strategy for increasing endogenous insulin production in T1D patients. Another area of focus is immunomodulatory therapies, which aim to address the autoimmune processes underlying T1D. By exploring the genetic factors contributing to the disease, researchers hope to develop therapies to prevent or delay the onset of T1D, ultimately improving patient outcomes [44]. The shift toward personalized medicine in treating T1D emphasizes tailoring therapies based on individual genetic, phenotypic, and lifestyle factors. A key component of this approach is the identification of biomarkers that can indicate disease progression and treatment response. Understanding these biomarkers will enable healthcare providers to develop personalized treatment plans that optimize efficacy while minimizing side effects [45]. Additionally, personalized medicine may involve combining different pharmacological agents based on a patient’s unique profile, ensuring that each individual receives the most appropriate therapy. Engaging patients in their treatment plans and utilizing technology for real-time monitoring and adjustments can lead to better outcomes and increased adherence. As research continues to advance, integrating personalized medicine approaches holds great promise for improving the management of T1D and enhancing the quality of life for patients [45].

Integrated care models

MDTs in healthcare consist of professionals from various disciplines collaborating to provide comprehensive patient care. This approach enhances treatment by integrating diverse expertise, which is particularly beneficial for managing complex health issues [46]. Key components of effective MDTs include collaboration, where members work together to develop and implement patient-centered treatment plans, ensuring that all aspects of a patient's health are addressed. This teamwork is crucial for improving patient outcomes and satisfaction. Role clarity is also important, as each team member has defined roles that leverage their specific skills, facilitating efficient care delivery. Understanding each other's strengths and responsibilities enhances communication and reduces the likelihood of errors. MDTs provide a more holistic approach, addressing medical needs and psychosocial aspects of patient care. This is particularly important in chronic disease management, where multiple health issues may coexist [46]. Patient education and self-management are integral to effective healthcare delivery, particularly within integrated care models. Empowering patients with knowledge about their conditions enables them to actively participate in their health management. Key strategies include educational programs, where patients are provided information about their conditions, treatment options, and self-care techniques, enhancing their understanding and adherence to treatment plans [47]. Goal setting is another important aspect, as encouraging patients to set personal health goals fosters accountability and motivation. This collaborative approach allows healthcare teams to align their support with the patient's aspirations. Establishing support networks, including family involvement and peer support groups, can enhance patient engagement and self-management capabilities [47].

Technology plays a crucial role in enhancing healthcare delivery and patient engagement. Telemedicine allows patients to consult healthcare providers remotely, improving access to care, especially for those in rural or underserved areas. Benefits include convenience, as patients can receive care without traveling, which is particularly beneficial for those with mobility issues or chronic conditions requiring frequent visits [48]. Telemedicine also facilitates ongoing communication between patients and providers, ensuring that care is consistent and responsive to changing health needs. Mobile health applications empower patients to manage their health actively. Features often include health tracking, where patients can monitor vital signs, medication adherence, and lifestyle choices, providing valuable data for patients and healthcare providers. Many apps offer educational materials and resources that help patients understand their conditions and treatment options, promoting informed decision-making. These apps often include messaging features that allow patients to communicate directly with their healthcare teams, enhancing coordination and support [48]. These advancements in MDTs, patient education, and technology integration collectively contribute to more efficient, effective, patient-centered healthcare delivery. By leveraging the strengths of diverse healthcare professionals, empowering patients through education and goal-setting, and utilizing innovative technologies, healthcare systems can better address the complexities of chronic disease management and improve overall patient outcomes.

Challenges and barriers to implementation

One of the primary barriers to effective CVD prevention in T1D is the fragmentation of care. Managing T1D often involves multiple healthcare providers, including endocrinologists, primary care physicians, dietitians, and cardiologists. This lack of coordinated care can lead to inconsistent messaging, missed opportunities for comprehensive risk assessment, and inadequate management of cardiovascular risk factors [49]. Additionally, many patients may not have access to specialized diabetes care or cardiology services, particularly in rural or underserved areas, which can hinder timely screening and management. Furthermore, healthcare providers may lack adequate training in the specific cardiovascular risks associated with T1D, and limited resources for patient education can impede effective communication about the importance of CVD prevention. Insurance coverage for preventive measures, such as routine screenings and medications, can also vary significantly, with high out-of-pocket costs discouraging patients from seeking necessary care or adhering to prescribed treatments [49]. Patient adherence and compliance present significant challenges in the management of T1D and the prevention of CVD. The complexity of diabetes management, which includes insulin therapy, blood glucose monitoring, dietary management, and physical activity, can overwhelm patients and lead to poor adherence to treatment regimens. Psychological factors, such as depression and anxiety, are prevalent among individuals with T1D and can significantly impact their ability to adhere to both diabetes management and cardiovascular prevention strategies [50]. Many patients may struggle with motivation and self-care, further complicating their health outcomes. Moreover, a lack of understanding of the relationship between diabetes and cardiovascular health can lead to non-compliance, as patients may not fully grasp the importance of managing risk factors or adhering to treatment plans. Lifestyle barriers, including time constraints and family responsibilities, can also interfere with patients' ability to make necessary lifestyle changes [50].

Socioeconomic and cultural factors are crucial in implementing CVD prevention strategies for individuals with T1D. Individuals from lower socioeconomic backgrounds often face barriers such as limited access to healthcare, nutritious food, and safe environments for physical activity, which can exacerbate cardiovascular risk. Cultural beliefs and attitudes toward diabetes and health can influence patients’ willingness to engage in preventive measures, with some cultures prioritizing alternative medicine or having different views on the importance of medication and lifestyle changes [51]. Health disparities are also significant, as racial and ethnic minorities often experience higher rates of diabetes and related complications, including CVD. Structural inequalities in healthcare access and quality contribute to these disparities, making it essential to address these issues to improve health outcomes. Finally, the availability of community resources, such as diabetes education programs and support groups, can vary widely, and a lack of community support can hinder patients' ability to make lifestyle changes and adhere to treatment plans [51]. Addressing these barriers requires a multifaceted approach. Enhancing coordination among healthcare providers through integrated care models and care teams can help ensure consistent messaging and comprehensive management of cardiovascular risks. Improving access to specialized care and patient education resources is critical, particularly in underserved areas. Training healthcare providers on the unique cardiovascular risks associated with T1D and the importance of preventive measures can improve patient care. Addressing insurance coverage gaps and reducing out-of-pocket costs for preventive care can encourage patients to seek necessary services. Supporting patients' psychological well-being and providing resources to improve adherence and compliance, such as diabetes education programs and support groups, can help manage the complexity of diabetes care. Lastly, addressing socioeconomic and cultural barriers through targeted interventions and community engagement can improve access to care and support for lifestyle changes.

## Conclusions

In conclusion, the prevention of CVD in individuals with T1D is of paramount importance due to the heightened risk and significant impact of CVD on morbidity and mortality in this population. This comprehensive review underscores the necessity of understanding the complex pathophysiological mechanisms linking T1D to CVD, recognizing the epidemiology and risk factors, and adhering to current risk assessment and screening guidelines. It emphasizes the effectiveness of lifestyle modifications, including dietary interventions, regular physical activity, and smoking cessation, alongside pharmacological interventions to optimize glycemic control, manage blood pressure, and improve lipid profiles. Additionally, emerging therapies and advances in insulin delivery systems present promising avenues for reducing CVD risk. The review also highlights the critical role of integrated care models, MDTs, and patient education in enhancing the management of T1D and preventing CVD. However, it acknowledges the challenges and barriers to implementing these strategies, such as healthcare system limitations, patient adherence issues, and socioeconomic factors. Addressing these challenges through targeted research, policy development, and clinical practice improvements is essential for reducing the burden of CVD in individuals with T1D and improving their overall health outcomes.
